# Evaluation of gut modulatory and bronchodilator activities of *Amaranthus spinosus* Linn.

**DOI:** 10.1186/1472-6882-12-166

**Published:** 2012-10-01

**Authors:** Mueen Ahmad Chaudhary, Imran Imran, Samra Bashir, Malik Hassan Mehmood, Najeeb-ur Rehman, Anwarul-Hassan Gilani

**Affiliations:** 1 Faculty of Pharmacy, Bahauddin Zakariya University, Multan, Pakistan; 2 Natural Product Research Unit, Department of Biological and Biomedical Sciences, Aga Khan University Medical College, Karachi, 74800, Pakistan

**Keywords:** *Amaranthus spinosus*, Laxative, Spasmolytic, Bronchodilator, Cholinergic, Ca^++^ antagonist

## Abstract

**Background:**

The aqueous-methanolic extract of *Amaranthus spinosus* (*A. spinosus* Linn.,) whole plant, was studied for its laxative, spasmolytic and bronchodilator activities to validate some of its medicinal uses.

**Methods:**

The crude extract of *A. spinosus* was studied *in-vivo* for bronchodilator and laxative activities and *in-vitro* using isolated tissue preparations which were mounted in tissue baths assembly containing physiological salt solutions, maintained at 37°C and aerated with carbogen, to assess the spasmolytic effect and to find out the possible underlying mechanisms.

**Results:**

In the *in-vivo* experiments in mice, the administration of *A. spinosus* increased fecal output at doses of 100 and 300 mg/kg showing laxative activity. It also inhibited carbachol-induced bronchospasm in anesthetized rats at 1, 3, 10 and 30 mg/kg indicative of bronchodilator activity. When tested on isolated gut preparations, the plant extract showed a concentration-dependent (0.01-10.0 mg/ml) spasmogenic effect in spontaneously contracting rabbit jejunum and guinea-pig ileum. The spasmogenic effect was partially blocked in tissues pretreated with atropine (0.1 μM). When tested on K^+^ (80 mM)-induced sustained contractions in isolated rabbit jejunum, the plant extract caused complete relaxation and also produced a shift in the Ca^++^ concentration-response curves (CRCs) towards right, similar to diltiazem. In rabbit trachea, the plant extract completely inhibited K^+^ (80 mM) and carbachol (CCh, 1 μM)-induced contractions at 1 mg/ml but pretreatment of tissue with propranolol (1 μM), caused around 10 fold shift in the inhibitory CRCs of the plant extract constructed against CCh-induced contraction. The plant extract (up to 0.3 mg/ml) also increased both force and rate of spontaneous contractions of isolated guinea-pig atria, followed by relaxation at higher concentration (1.0-5.0 mg/ml). The cardio-stimulant effect was abolished in the presence of propranolol, similar to that of isoprenaline. Activity-directed fractionation revealed that the spasmolytic component(s) was separated in the organic fraction, whereas the spasmogenic component was concentrated in the aqueous fraction.

**Conclusion:**

These results indicate that *A. spinosus* possesses laxative activity partially mediated through cholinergic action. The spasmolytic effect was mediated through calcium channel blocking (CCB), while bronchodilator activity through a combination of β-adrenergic and CCB pathways, which may explain the traditional uses of *A. spinosus* in gut and airways disorders.

## Background

*Amaranthus spinosus* Linn. (Family, Amaranthaceae), commonly known as Prickly amaranth and locally in Paksitan as Khaddar-chaulai, is a spinous weed cultivated throughout tropical and subtropical countries for its use as vegetable and also as an animal feed [1,2]. The plant is highly nutritive due to the presence of fibre, proteins and high concentration of essential amino acids, especially lysine [3]. Besides its culinary value, it is a popular medicinal plant reputed for antipyretic, appetizer, diuretic, febrifuge, galactagogue, haematinic, laxative and stomachic effects and as a treatment for hallucination, leprosy, eczema and piles [2]. It is also useful in bronchitis, leucorrhoea, menorrhagia, boils, burns, nausea, flatulence and colic [4], healing of wounds and rheumatism [5], and to arrest the coughing up of blood [6]. All parts of the plant are known to contain medicinally active constituents [1].

Different studies have evaluated *A. spinosus* for the presence of antioxidant [7], antinociceptive [8], hepato-protective [9], anti-diabetic, anti-hyperlipidemic, spermatogenic [10] and antimalarial [11] activities.

Phytochemical studies revealed that the plant contains alkaloids, flavonoids, glycosides, phenolic acids, steroids, amino acids, terpenoids, lipids, saponins, betalains, β-sitosterol, stigmasterol, linoleic acid, rutin, catechuic tannins and carotenoids [9]. The betalains in stem bark of *A. spinosus* were identified as amaranthine, isoamaranthine, hydroxycinnamates, quercetin and kaempferol glycosides [11-15]. It also contains amaranthoside, a lignan glycoside, amaricin, a coumaroyl adenosine along with stigmasterol glycoside, betaine such as glycinebetaine and trigonelline [16,17].

Despite the wide medicinal uses of *A. spinosus*, no data are available with respect to its effectiveness in gut motility and airways disorders. The present study on the crude extract of *A. spinosus* and its fractions was undertaken to rationalize these traditional uses and to explore mechanistic basis for these medicinal uses.

## Methods

### Plant material

Fresh whole plant of *A. spinosus* Linn. was collected from the fields of Bahauddin Zakariya University Multan, Pakistan, and was identified by an expert taxonomist of the Institute of Pure and Applied Biology, Bahauddin Zakariya University, Multan. A specimen is deposited in Herbarium of the Institute (voucher # Fl.P.231-2).

### Preparation of crude extract and fractions

Following shade drying, the plant material was manually made free from soil and other adulterants and was ground to coarse powder by an electrically driven mill. Approximately 400 g powdered plant material was soaked in 70% aqueous-methanol by cold maceration at room temperature for 7 days with occasional shaking [18]. It was filtered through a double layered muslin cloth and subsequently through a filter paper. The residue was re-soaked in the fresh solvent and the process was repeated thrice to get maximum yield of crude extract from the plant material. The combined filtrate was concentrated in rotary evaporator at 40°C under reduced pressure (−760 mmHg) to a thick, semi-solid mass, the crude extract of *A. spinosus* whole plant (As.Cr), weighing 44.52 gm with approximated yield of 11.13%. *A. spinosus* was transfer to a glass bottle and stored at −20°C until used. The stock solutions of *A. spinosus* were prepared in distilled water and the dilutions were made fresh in normal saline on the day of experiment.

For the purpose of fractionation, 10 gm of the crude extract was dissolved in water and shaken with ethyl acetate in a separating funnel. Two layers of the immiscible solvents were allowed to separate. The ethyl acetate layer was collected whereas the aqueous layer was re-extracted with fresh ethyl acetate for a total of three times. The ethyl acetate layers, so collected, were combined and then dried on a rotary evaporator to yield the ethyl acetate fraction (As.EtAc) weighing 0.3 gm. The aqueous layer left after extraction with ethyl acetate was evaporated separately to obtain the aqueous fraction (As.Aq) weighing 9.7 gm. Approximate yields of As.EtAc and As.Aq were 3% and 97% of the crude extract, respectively. The As.EtAc was solubilized by the following method; a solution of Tween-80 (100 mg/ml in ethyl acetate) was prepared, and 0.1 ml of this solution was added to the solution of As.EtAc (300 mg/ml) in ethyl acetate and shaken. The solvent was then evaporated on a hot plate and the resultant extract was suspended in water (1 ml) by vigorous shaking. The concentration of this stock is considered as 300 mg/ml [19].

### Drugs and reagents

All the chemicals used in the experiments were of highest purity and research grade and were obtained from the sources specified: acetylcholine chloride, atropine sulfate, aminophylline, carbachol, diltiazem, histamine, isoprenaline, pyrillamine, potassium chloride and phenylephrine from Sigma Chemicals Co. St Louis, MO, USA, calcium chloride, glucose, magnesium chloride, magnesium sulphate, potassium dihydrogen phosphate, sodium bicarbonate, sodium dihydrogen phosphate and methanol from Merck, Darmstadt, Germany, and ammonium hydroxide, sodium chloride and sodium hydroxide from BDH Laboratory Supplies, Poole, England.

### Animals

Rabbits (1.0-1.5 kg), guinea-pigs (500-600 g) and BALB/c mice (20-30 g) of local breed and either sex, used for the experimental work, were housed under controlled environmental condition (23-25°C) at the Animal House of Aga Khan University, Karachi. Animals were given standard diet and tap water. Animals were kept at fasting 24 h prior to the experiments but had free access to water. Rabbits and guinea-pigs used for *in-vitro* study were sacrificed by blow on the back of head and cervical dislocation, respectively. Mice were used for the *in-vivo* laxative activity. Experiments performed complied with the rulings of Institute of Laboratory Animal Resources, Commission on Life Sciences [20] and approved by the Ethical Committee of the Aga Khan University.

### *In-vivo* experiments

#### Laxative activity

Mice were used as experimental animals to study the laxative activity of *A. spinosus* in comparison with carbachol, by following a previously described method [21]. The food supply was restricted to the mice 24 h prior to the experiment but there was a free access to water. The animals, kept individually in cages lined with clean blotting sheets, were divided into five groups containing five mice each to receive different treatments, orally. Group 1, taken as the negative control, was administered with normal saline (10 ml/kg), Group 2, the positive control group, was administered with carbachol (1 mg/kg), while the Groups 3, 4 and 5, the test groups, received 100, 300 & 500 mg/kg of the crude extract of *A. spinosus*, respectively. All five groups were monitored for 18 h; total number of feces was counted for each mouse and the average number of feces was calculated for each group.

#### Effect on CCh-induced bronchospasm

Rats were anaesthetized with sodium thiopental (Pentothal, 80–100 mg/kg, i.p.) and underwent endotracheal intubation followed by ventilation with a volume ventilator (Miniature ideal pump, Bioscience, UK) adjusted at a rate of 70–80 strokes/min to deliver 7–10 ml/kg of room air [22]. A polyethylene catheter was inserted into the jugular vein for drug administration. The changes in airways resistance (mmHg) were measured by a pressure transducer (MLT-1199) connected to PowerLab 4/25 with running chart software via Quad bridge amplifier (AD Instruments, Bella Vista, NSW, Australia). Bronchoconstriction was induced with CCh (100 μg/kg), which was reversed within 7–10 min. The test drug was given to the animals 5–8 min prior to administration of CCh. The responses were expressed as the percent reduction of the CCh-induced bronchospasm [22].

### *In-vitro* experiments

The *in-vitro* experiments were performed according to the protocols as previously described [23-25].

#### Rabbit jejunum

Plant extract was screened for its spasmogenic and spasmolytic activities on isolated rabbit jejunum. Segments of approximately 2 cm length were suspended in a 10 ml tissue bath containing Tyrode’s solution having the following composition in mM: KCl 2.68, NaCl 136.9, MgCl_2_ 1.05, NaHCO_3_ 11.90, NaH_2_PO_4_ 0.42, CaCl_2_ 1.8 and glucose 5.55, aerated with carbogen (95% O_2_ and 5% CO_2_) and maintained at 37°C. Each tissue was given 1 g pretension, allowed to equilibrate for at least 30 min and stabilized with the repeated exposure to 0.3 μM acetylcholine, ACh (3–5 times) and subsequent washing with the Tyrode’s solution until the sub-maximal responses of equal amplitude were obtained. The dose–response curves of acetylcholine were constructed before the addition of test materials. Maximum response of the tissue to acetylcholine was considered to have been achieved when the next higher concentrations of the agonist failed to produce a further increase in response [26]. The contractile effect of the test materials was assessed as percent of the maximum effect produced by the control drug, ACh. Intestinal responses were obtained isotonically using BioScience transducers and Powerlab data acquisition system (AD Instruments, Sydney, Australia) attached to a computer installed with Labchart software (version 6).

#### Determination of Ca^++^ antagonist effect

To assess whether the relaxant effect of the crud extract was through calcium channel blocking activity, K^+^ (80 mM) was used to depolarize the preparations as described by Farre et al. [27]. K^+^ (80 mM) was added to induce the sustained contraction. The crud extract was then added to the tissue bath in a cumulative fashion to obtain concentration-dependent inhibitory responses [28]. The relaxation of intestinal preparations, pre-contracted with K^+^ (80 mM) was expressed as percent of the control response mediated by K^+^. To confirm the calcium antagonist activity of the crud extract, the tissue was allowed to stabilize in normal Tyrode’s solution, which was then replaced with Ca^++^-free Tyrode’s solution containing EDTA (0.1 mM) for 30 min in order to remove Ca^++^ from the tissues. This solution was further replaced with K^+^-rich and Ca^++^-free Tyrode’s solution, having the following composition in mM: KCl 50, NaCl 91.04, MgCl_2_ 1.05, NaHCO_3_ 11.90, NaH_2_PO_4_ 0.42, glucose 5.55 and EDTA 0.1. Following an incubation period of 30 min, control concentration–response curves of Ca^++^ were constructed. When the control CRCs of Ca^++^ were found super-imposable (usually after two cycles), the tissue was pretreated with the crude extract for 50 min to test the possible calcium channel blocking effect. The CRCs of Ca^++^ were reconstructed in the presence of different concentrations of the test material, diltiazem was used as a positive control.

#### Guinea-pig ileum

The ileum was dissected out and segments of approximately 2 cm length were suspended individually in a 10 ml tissue bath, filled with Tyrode’s solution and aerated with carbogen at 37°C [29]. A preload of 1 g was applied to each tissue and kept constant throughout the experiment. Following an equilibration period of 30 min, isotonic contractions to ACh (0.3 μM) were repeated to stabilize the preparation. The stimulant effect of the extract was determined on the resting baseline of the tissue and was assessed as percent of the maximum effect produced by the reference drug, ACh.

#### Rabbit trachea

Trachea was dissected out, cleaned free from the surrounding fatty tissues and cut into rings having 2–3 mm width (containing 2 cartilages). Each ring was then cut longitudinally on the site opposite to the smooth muscle layer in such a way that smooth muscles were in between the C-shaped cartilaginous part. The isolated preparations were then mounted in a 15 ml tissue bath containing Kreb’s solution having the following composition in mM: NaCl 118.2, NaHCO_3_ 25.0, CaCl_2_ 2.5, KCl 4.7, KH_2_PO_4_ 1.3, MgSO_4_ 1.2 and glucose 11.7, aerated with carbogen at 37°C. The mounted tissue preparations were given 1 g pre-tension and equilibrated for 1 h before the addition of any chemical substance. To induce sustained contractions, carbachol (1 μM) and high K^+^ (80 mM) were used and the bronchodilator activity was studied by adding plant extract in a cumulative manner. Isometric responses were obtained with the help of BioScience transducers and recorded through PowerLab data acquisition system [30].

#### Guinea-pig atria

Right atrium from the healthy guinea-pig was dissected out, cleaned off fatty tissues and mounted in a 15 ml tissue bath containing Kreb’s solution aerated with carbogen at 32°C [31]. The tissue exhibited spontaneous beating under the resting tension of 1 g due to the presence of pacemaker cells. An equilibrium period of 30 min was provided before the application of any chemical substance. This preparation allowed us to study the effect of the test substance on both rate and force of spontaneous atrial contractions. The data obtained represent the force of atrial contractions, while rate of atrial contractions was obtained by increasing speed of the chart. The control responses of isoprenaline (1 μM) were obtained at least in duplicate. Isometric responses were obtained with the help of BioScience transducers and recorded through PowerLab data acquisition system.

### Statistical analysis

The data are expressed as mean ± standard error of the mean (S.E.M., n = number of experiments) or the median effective concentrations (EC_50_ values) given with 95% confidence intervals (CI). The statistical parameter applied is the Student’s *t*-test and One-way analysis of variance (ANOVA) followed by Dunnett’s test. *P* < 0.05 is considered as significant.

## Results

### *In vivo* experiments

#### Laxative activity

**Figure 1:**
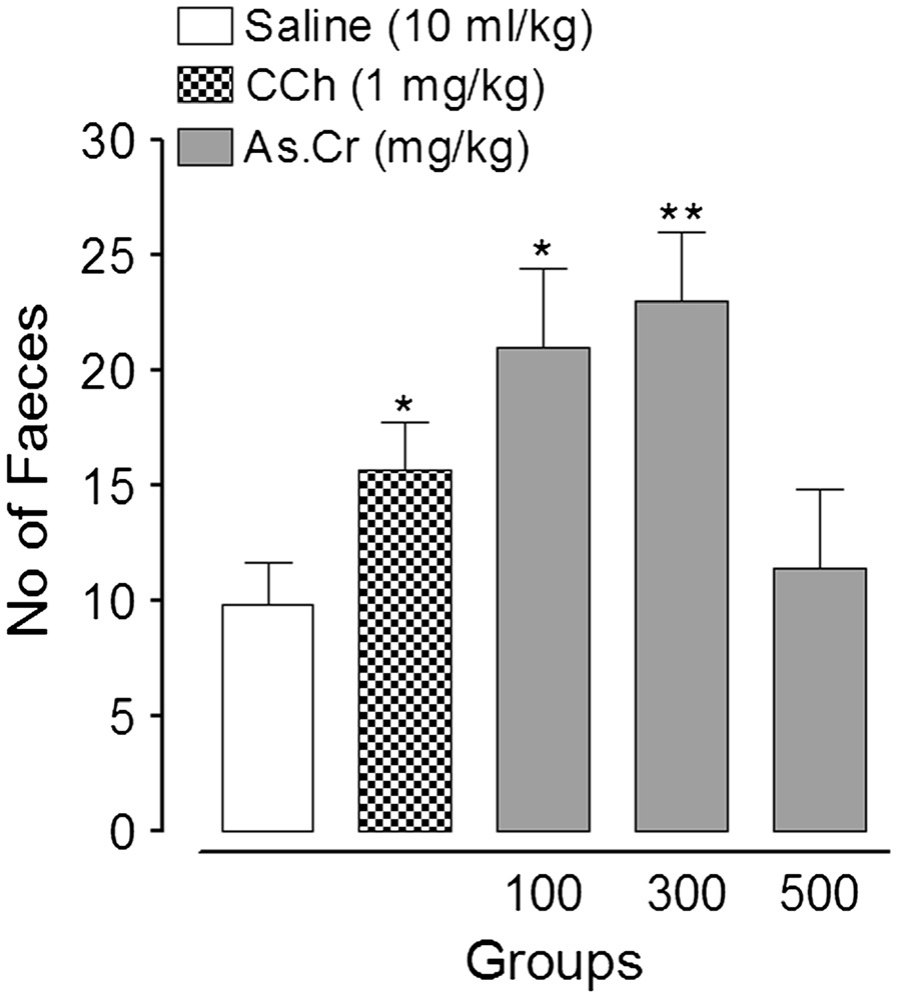
**Laxative effect of variousconcentrations of crude extractof*A. spinosus*(As.Cr) and carbachol (CCh)in healthy mice.** *p < 0.05, **p < 0.01 vs. Saline.

When administered orally, the plant extract showed laxative effect in mice as reflected by an increase in the total number of feces (Figure 1). The laxative effect was mediated at the dose level of 100 and 300 mg/kg and was 20.5 ± 3.1 and 23 ± 3.0, respectively, as opposed to 7.9 ± 1.3 feces with saline. Carbachol similarly increased the fecal output to 18.9 ± 1.5 at 10 mg/kg. The laxative effect of the plant extract was declined with further increase in dose to 500 mg/kg.

#### Bronchodilator activity

**Figure 2:**
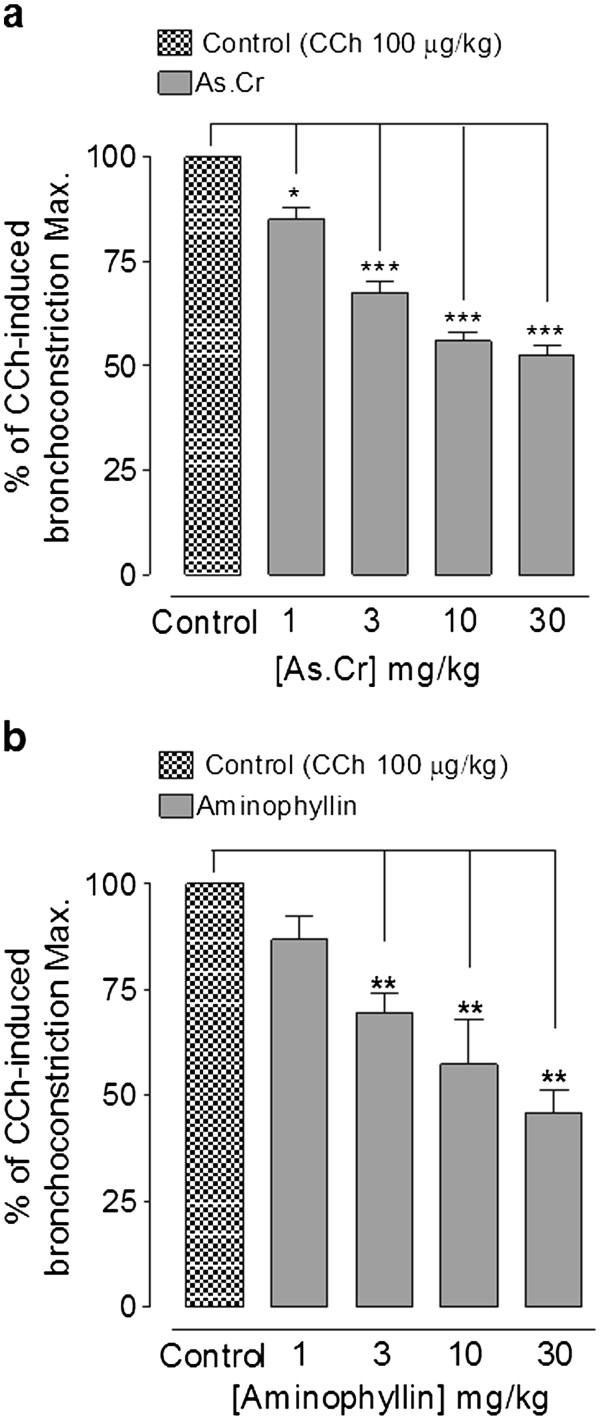
**Dose-dependent inhibitory effect of(a) the crude extractof*A. spinosus*(As.Cr) and (b) aminophyllineon carbachol (CCh)-induced bronchoconstrictionin anaesthetized rats.** Values shown are mean ± S.E.M, n = 4–6, *P < 0.05, **P < 0.01, ***P < 0.001 vs. control (CCh), One-way ANOVA, followed by Dunnett’s test.

The crude extract of *A. spinosus* at the doses of 1, 3, 10 and 30 mg/kg caused 15.0 ± 3.0, 32.5 ± 2.5, 44.0 ± 2.0 and 47.5 ± 2.5% (n = 4) respective suppression of CCh (100 μg/kg)-induced increase in respiratory pressure of anaesthetized rats (Figure 2a). Inhibitory effect of *A. spinosus* was comparable to aminophylline, used as a positive control, which inhibited the CCh-mediated bronchoconstriction at similar doses by 13.3 ± 5.8, 30.5 ± 4.5, 42.8 ± 10.6 and 54.1 ± 5.2% (n = 4), respectively (Figure 2b).

### *In vitro* experiments

#### Effect on rabbit jejunum

**Figure 3:**
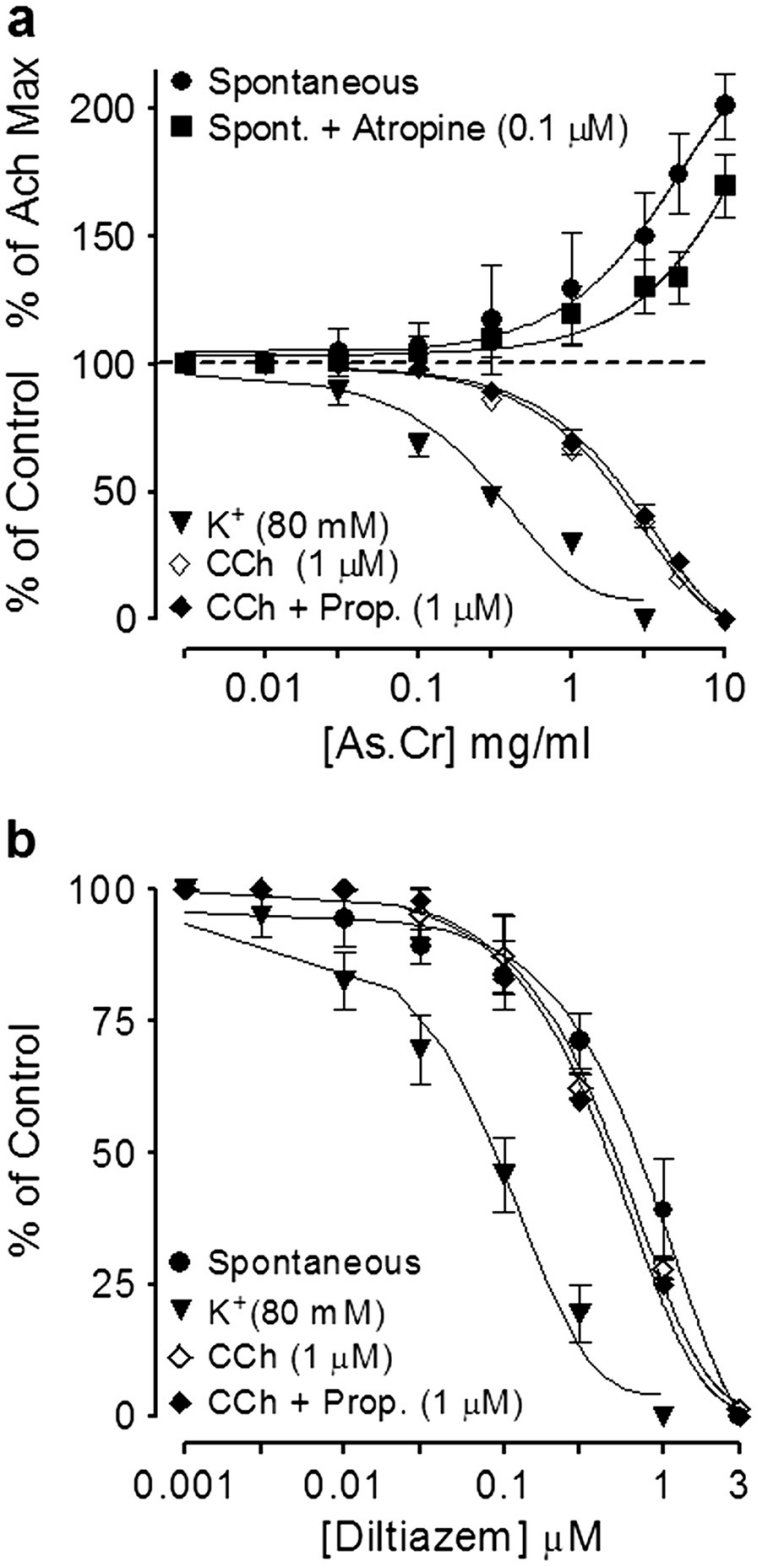
**Concentration-dependent effect of (a)the crude extract of*A. spinosus*(As.Cr) and (b) diltiazemon spontaneous, K**^**+**^**and carbachol (CCh)-induced contractionsin isolated rabbit jejunum.** The stimulant effect of As.Cr on spontaneous contraction is taken in the absence and presence of atropine (0.1 μM), while the inhibitory effect of both As.Cr and diltiazem against CCh-induced contractions is taken in the absence and presence of propranolol (Prop., 1 μM). Values shown are mean ± S.E.M., n = 3–5.

**Figure 4:**
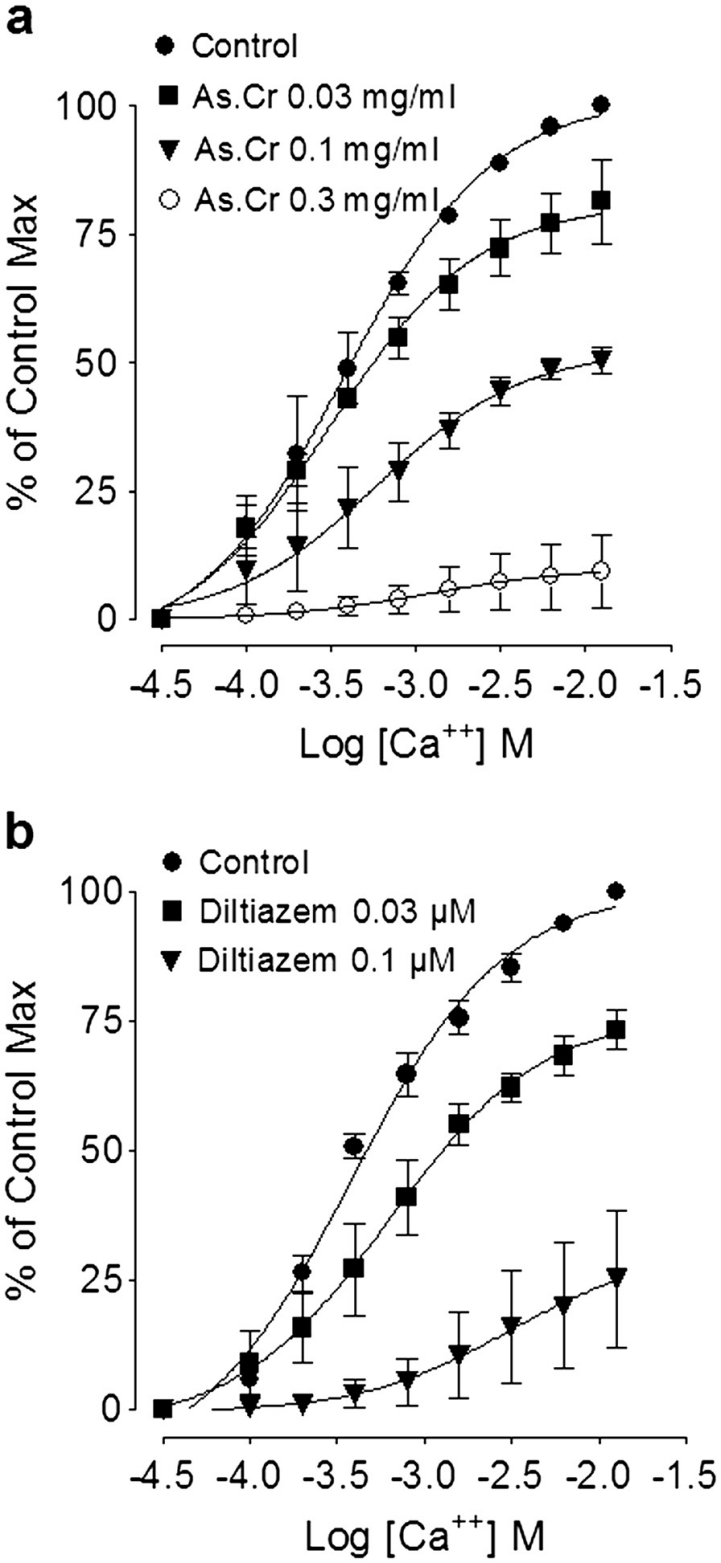
**Concentration-response curves of Ca**^**++**^**in the absence andpresence of (a) thecrude extract of*A. spinosus*(As.Cr) and (b) diltiazemin isolated rabbit jejunum.** Values are expressed as mean ± S.E.M., n = 4.

In spontaneously contracting rabbit jejunum preparations, the crude extract of *A. spinosus* showed a concentration-dependent (0.3-10 mg/ml) spasmogenic effect, which did not sustain and was subsequently followed by relaxation when the tissue was left unwashed for some time (Figure 3a). The spontaneous contractions of the tissue were fully revived after washing with Tyrode’s solution and the spasmogenic effect was reproducible. The spasmogenic effect, measured as % of ACh (3.0 μM)-induced maximum response was 0.57 ± 0.26, 1.1 ± 0.32, 3.60 ± 0.92, 9.02 ± 2.24, 21.00 ± 4.09, 44.39 ± 5.22, 72.57 ± 7.55 and 99.16 ± 6.14 (mean ± S.E.M., n = 4) at the respective concentrations of 0.01, 0.03, 0.1, 0.3, 1.0, 3.0, 5.0 and 10.0 mg/ml. Pre-treatment of the tissues with atropine (0.1 μM) partially suppressed the spasmogenic effect of the plant extract (Figure 3a). The plant extract also relaxed high K^+^ and CCh (1 μM)-induced contractions (Figure 3a) with respective EC_50_ value of 0.31 (0.22-0.46, 95% CI, n = 5) and 2.3 mg/ml (0.17-2.61, n = 5), similar to diltiazem, which relaxed spontaneous, high K^+^ and CCh-induced contractions with respective EC_50_ values of 0.69 (0.58-0.83, n = 3), 0.09 (0.07-0.11, n = 4) and 0.63 mg/ml (0.56-0.72, n = 4) (Figure 3b). The relaxant effect of both the plant extract and diltiazem against CCh-induced contraction was not affected by pretreatment of the tissues with propranolol (1 μM), however, pretreatment of tissue with the crude extract of *A. spinosus* shifted the Ca^++^ concentration-response curves (CRCs) rightwards in a concentration-dependent manner (0.03-0.3) similar to diltiazem (0.03 and 0.1 μM), as shown in Figure 4a and b, respectively.

#### Effect on guinea-pig ileum

**Figure 5:**
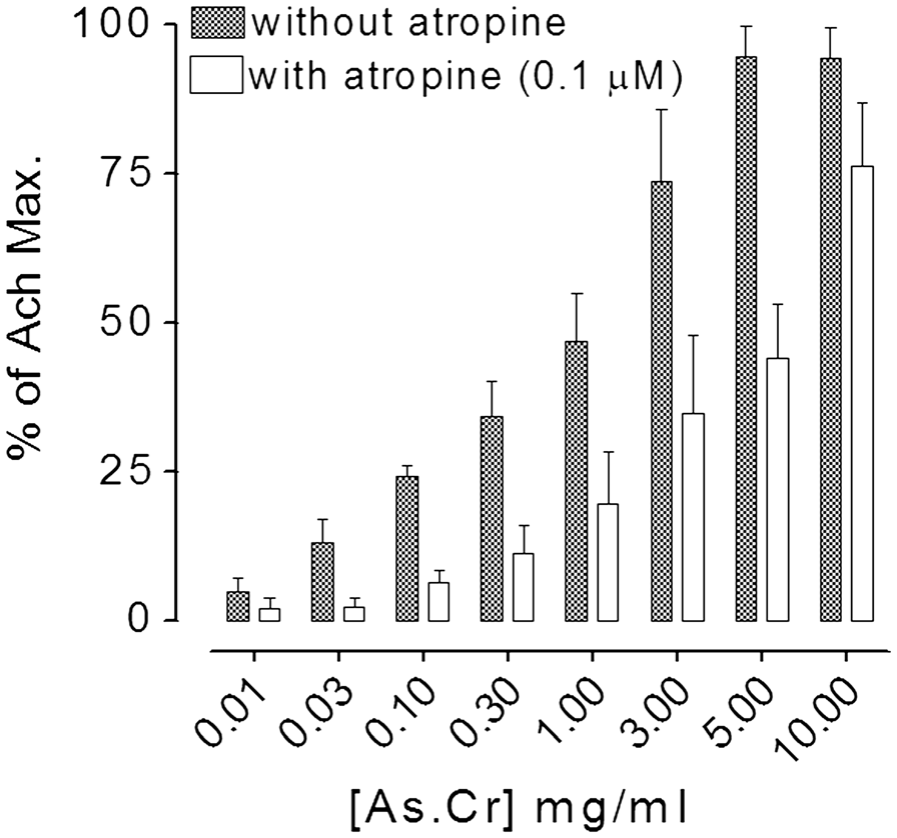
**Bar-chart showing the concentration-dependentspasmogenic effect of thecrude extract of*A. spinosus*(As.Cr) in the absenceand presence of atropine(0.1 μM) in isolatedguinea-pig ileum.** The responses are shown as % of Ach (1 μM)-induced maximum contractile effect. Values are given as mean ± S.E.M., n = 4–5.

The crude extract of *A. spinosus* caused a concentration-dependent (0.01-10 mg/ml) spasmogenic effect in isolated guinea-pig ileum (Figure 5). The spasmogenic effect, measured as % of ACh (1 μM)-induced maximum contraction, was 4.89 ± 2.29, 12.99 ± 4.07, 24.21 ± 1.85, 34.29 ± 5.89, 46.87 ± 8.00, 73.65 ± 12.09, 94.61 ± 5.20, and 94.41 ± 5.10 (n = 4) at the respective concentration of 0.01, 0.03, 0.1, 0.3, 1, 3, 5, and 10 mg/ml, showing maximum response to that of ACh. The spasmogenic effect of crude extract was partially suppressed in tissues pre-treated with atropine (0.1 μM) as shown in Figure 5.

#### Effect on rabbit trachea

**Figure 6:**
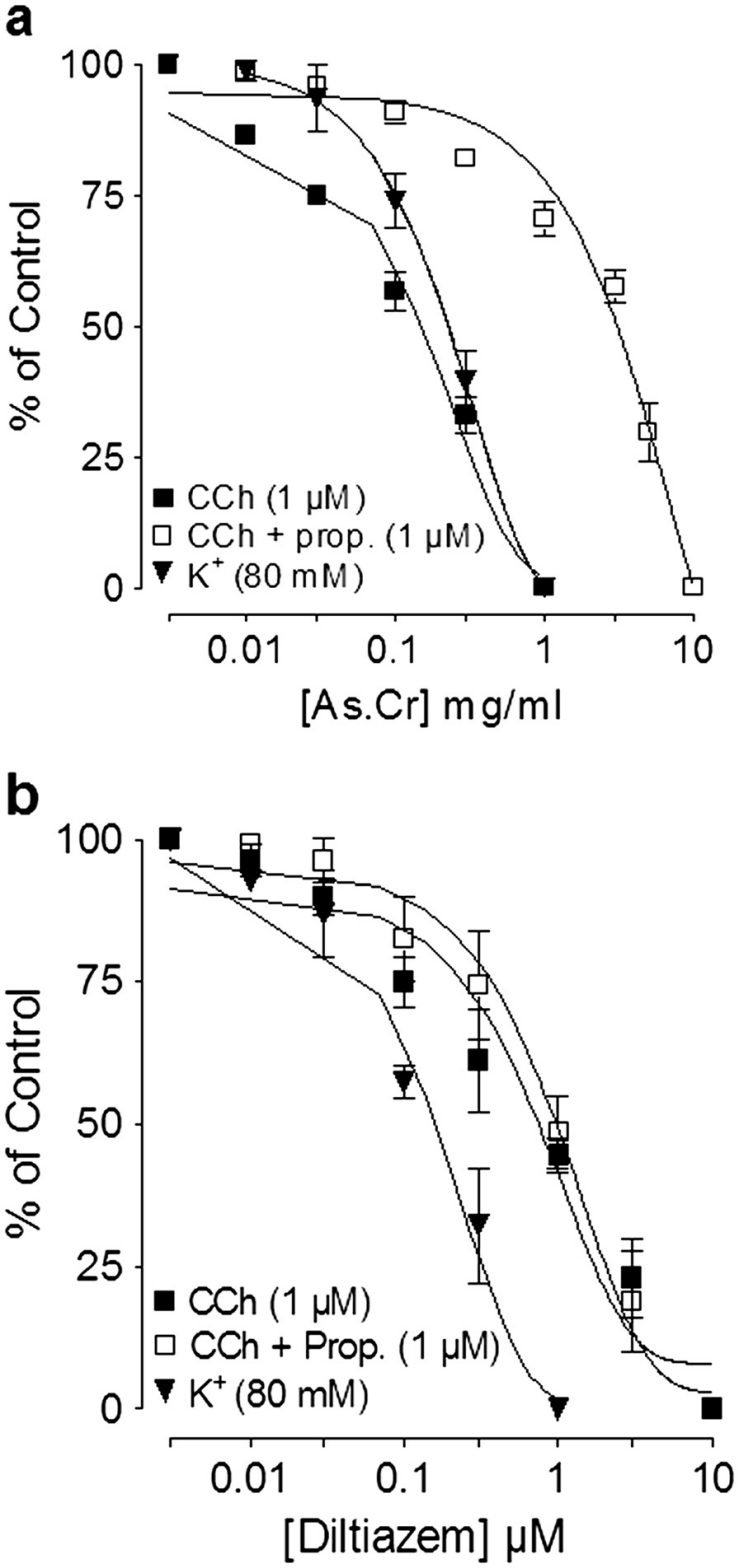
**Concentration-dependent relaxation effects of(a) the crude extractof*A. spinosus*(As.Cr) and (b) diltiazemon K**^**+**^**and CCh-induced contractions inisolated rabbit trachea.** Effect of As.Cr on CCh-induced contraction is recorded in the presence and absence of propranolol (prop). Values are shown as mean ± S.E.M., n = 3–6.

**Figure 7:**
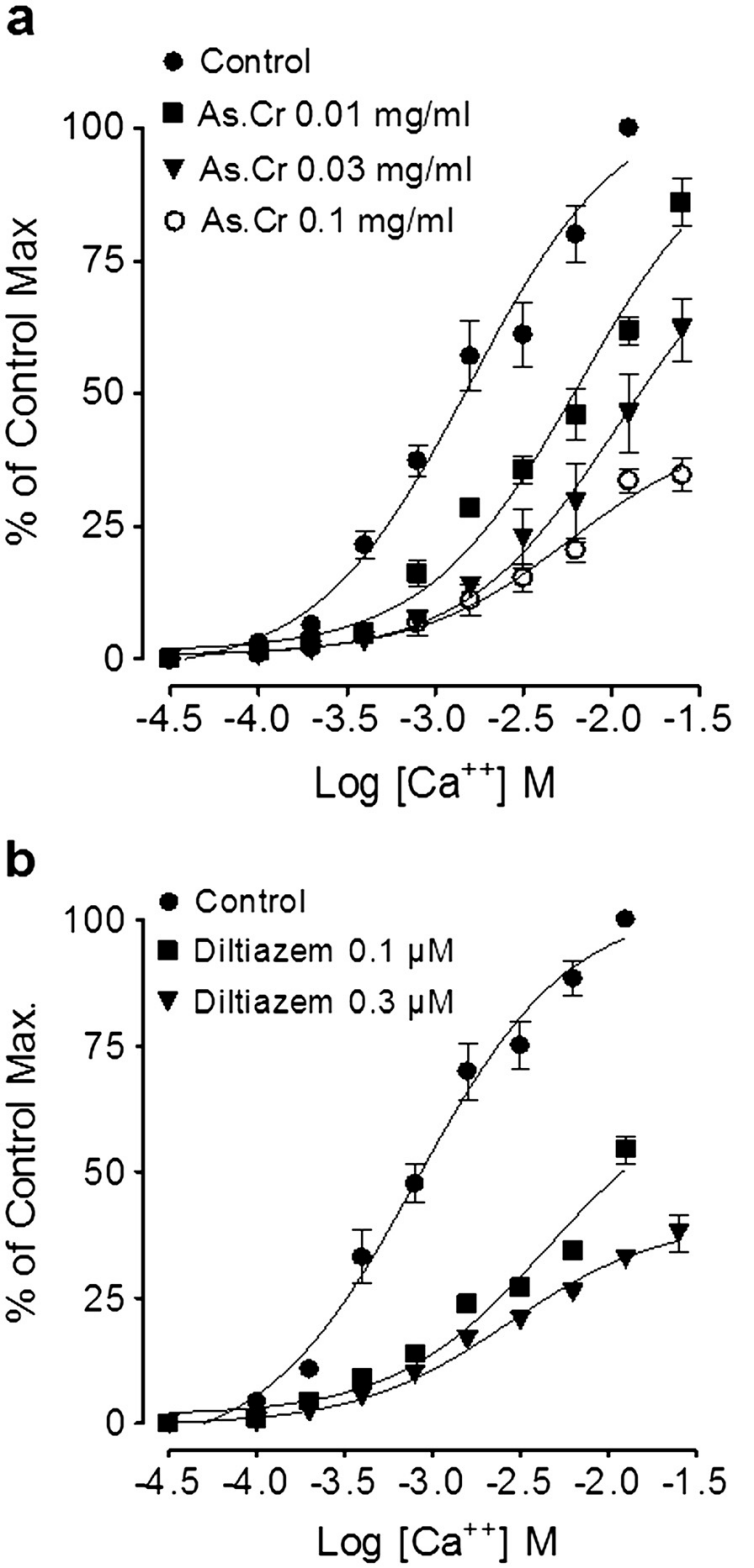
**Concentration-response curves of Ca**^**++**^**in the absence andpresence of (a) thecrude extract of*A. spinosus*(As.Cr) and (b) diltiazemin isolated rabbit trachea.** Values are expressed as mean ± S.E.M., n = 3–5.

When tested on isolated rabbit trachea, the plant extract inhibited K^+^ (80 mM) and CCh (1 μM)-induced contractions (Figure 6a) with respective EC_50_ values of 0.24 mg/ml (0.18-0.30, n = 5) and 0.15 mg/ml (0.10-0.22, n = 5). Pretreatment of the tissues with propranolol (1 μM) cause a rightward shift in the inhibitory CRCs of the plant extract constructed against CCh-induced contraction with resultant EC_50_ value of 3.23 mg/ml (2.51-4.15, n = 4). Diltiazem inhibited high K^+^ and CCh (1 μM)-induced contractions with EC_50_ values of 0.22 mg/ml (0.14-0.39, n = 4) and 0.72 mg/ml (0.52-1.0, n = 4), respectively (Figure 6b), but the relaxant effect of diltiazem was not affected by the presence of propranolol. When tested on Ca^++^ curves, the plant extract concentration-dependently (0.01-0.1 mg/ml), shifted Ca^++^ CRCs towards right similar to diltiazem (0.1 and 0.3 μM), as shown in Figure 7a and b, respectively.

#### Effect on guinea-pig atria

**Figure 8:**
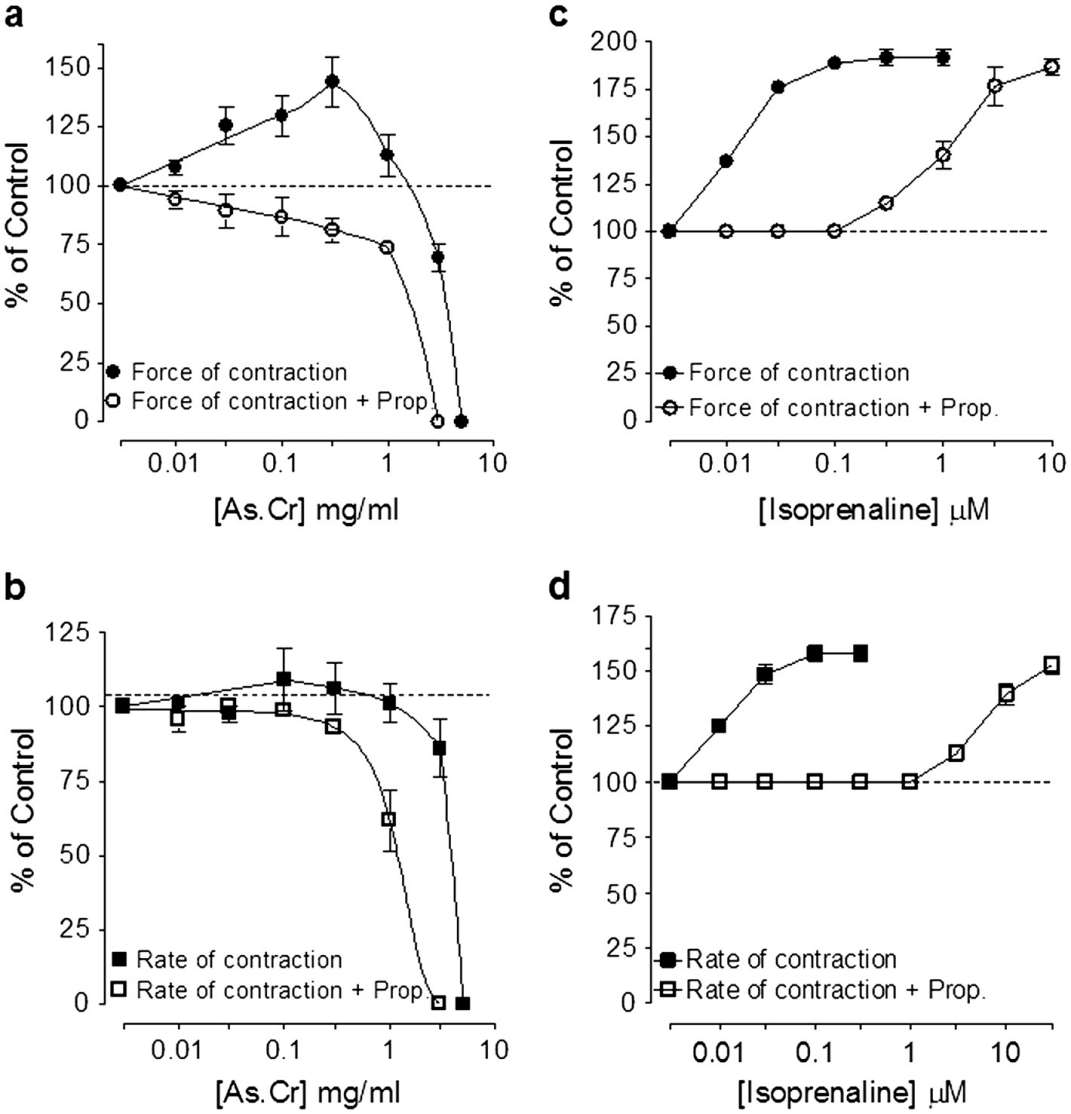
**Effect of (a andb) the crude extractof*A. spinosus*(As.Cr) and (c andd) isoprenaline in theabsence and presence ofpropranolol (Prop.,1 μM) onforce and rate ofspontaneous contractions of isolatedguinea-pig right atria.** Values are shown as mean ± S.E.M., n = 3–5.

When tested on spontaneously beating guinea-pig atria, the crude extract of *A. spinosus* increased both force and rate of contraction at 0.01-0.3 mg/ml, similar to isoprenaline (0.01-1 μM), as shown in Figure 8. With further increase in concentrations, the plant extract did not cause further increase in cardiac contractility (Figure 8a). The cardio-stimulant effect of the plant extract was inhibited in the tissues pre-treated with propranolol (1 μM) as shown in Figure 8a. The effect on heart rate was found blunt at lower concentration (0.01-0.3 mg/ml), while dose-dependent inhibitory effect was observed at higher concentrations (1.0-5.0 mg/ml). Similar to the inotropic effect, the chronotropic effect was also inhibited in the presence of propranolol (Figure 8b). Similarly, the cardiostimulant effect of isoprenaline (0.01-0.3 μM) both on force and rate of contraction were shifted towards right when reproduced in the tissues pretreated with propranolol (Figure 8c and d).

#### Effect of fractions on rabbit jejunum

**Figure 9:**
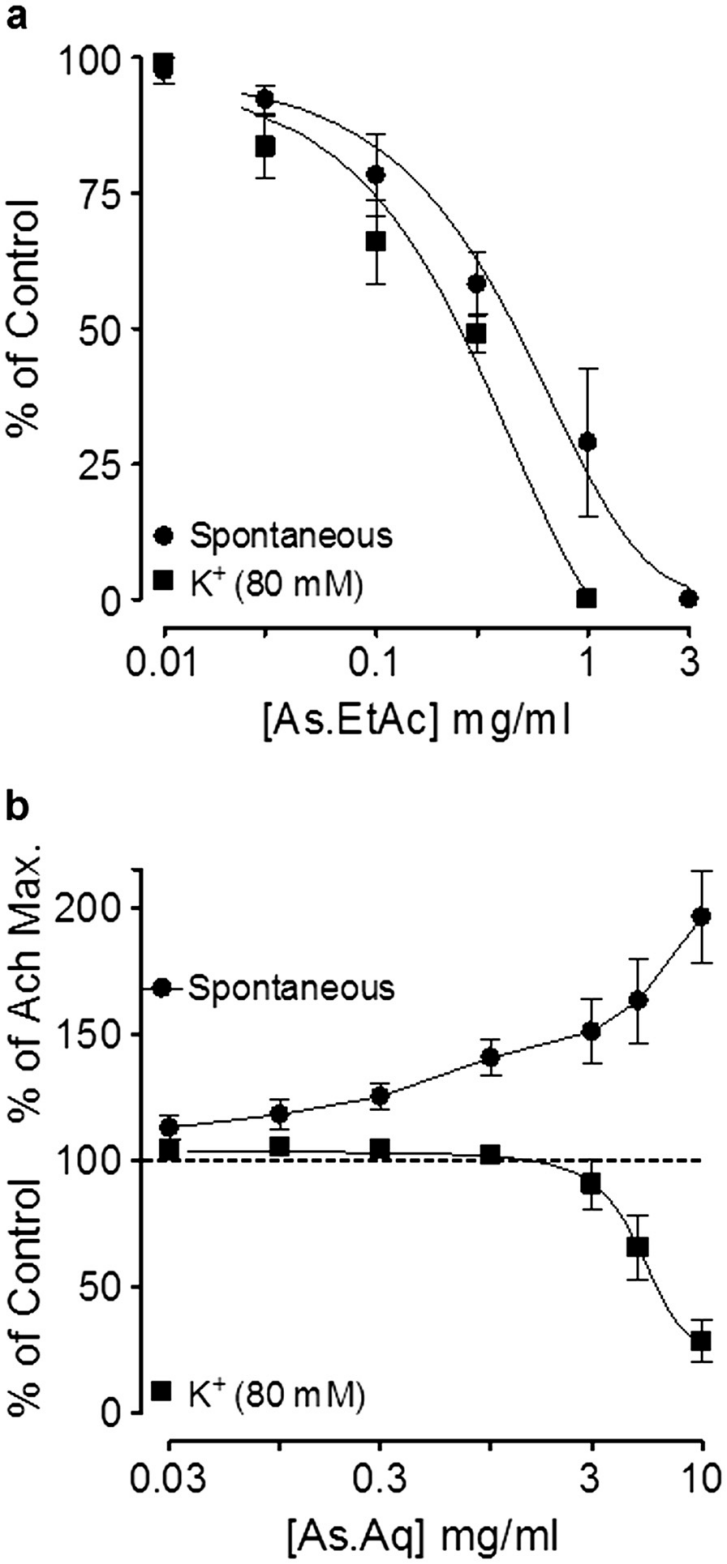
**Concentration-dependent effect of (a)ethyl acetate (As.EtAc) and(b) aqueous (As.Aq) fractionsof*Amaranthus spinosus*on spontaneous and K**^**+**^**(80 mM)-induced contractions inisolated rabbit jejunum.** Values shown are mean ± S.E.M., n = 4–5.

The ethyl acetate fraction of the crude extract of *A. spinosus* (As.EtAc) was found devoid of stimulant effect and exhibited concentration-dependent relaxant effect on spontaneous and high K^+^ (80 mM)-induced contractions with EC_50_ values of 0.48 mg/ml (0.37-0.63, n = 4) and 0.27 mg/ml (0.19-0.38, n = 4), respectively (Figure 9a).

The aqueous fraction (As.Aq) produced a concentration-dependent spasmogenic effect at 0.01-10.0 mg/ml (Figure 9b) with maximum spasmogenic effect of 96.28 ± 8.10% ; (n = 5) close to ACh-induced maximum response (*P >* 0.05), followed by relaxation when tissue was left unwashed for some time and showed weak inhibitory effect on K^+^ (80 mM)-induced contraction.

## Discussion

In view of its medicinal use in gastrointestinal and airways disorders, the crude extract of *A. spinosus* was subjected to pharmacological investigation in order to validate the medicinal uses and explore possible underlying mechanisms.

To observe its laxative effect, the plant extract was administered orally to healthy mice, which produced a dose-dependent laxation at 100 and 300 mg/kg, similar to carbachol (1 mg/kg). A further increment in dose (500 mg/kg) decreased the fecal output possibly due to the presence of spasmolytic component along with spasmogenic constituents. In addition, there are multiple reports showing the safety of plant administration in experimental animals at around 4 times higher doses than the highest dose tested in this study [32,33].

To explore the possible mechanism possibly underlying its laxative effect, the crude extract was tested on spontaneously contracting rabbit jejunum, where it showed a concentration-dependent spasmogenic effect followed by relaxation when the tissue was left unwashed for some time. The spontaneous movement of intestine is regulated by periodic depolarization and re-polarization and the peak of depolarization is represented by action potential generated due to the rapid influx of Ca^++^ via voltage dependant L-type Ca^++^ channels (VDLCs) [34]. Maximum spasmogenic effect of the crude extract was found similar to ACh-induced maximum contractile response suggesting the high efficacy of its spasmogenic effect. To see if the gut stimulant effect was mediated through cholinergic activity, the stimulant effect was challenged by atropine (0.1 μM), a known muscarinic receptor antagonist [35]. In the presence of atropine, the contractile effect of the extract was partly blocked, indicating the presence of cholinergic constituent(s) and some additional mechanism(s) responsible for the observed stimulant effect. The use of the specific antagonists also ruled out the possible presence of any histaminergic, serotonergic or nicotinic activity in the crude extract of *A. spinosus* mediating its spasmogenic effect (data not shown).

The gut stimulant effect of the crude extract was further studied on guinea-pig ileum, a quiescent preparation considered useful for this purpose [29]. The plant extract exhibited dose dependent contractions in guinea-pig ileum partly suppressed by atropine, a similar pattern of activity was observed in jejunum thus confirming the involvement of more than one pathway in the spasmodic effect of the plant extract. Results of this study indicate the presence of spasmogenic constituents in the plant that may account for its medicinal use in constipation and also invite further experimental work to explore the exact nature of additional spasmogenic component.

The spasmogenic effect in gut preparations was followed by spontaneous relaxation when the tissues were left unwashed for some time, indicating the presence of spasmolytic component in combination with spasmogenic activity. In our previous studies we have noticed the presence of Ca^++^ channel blocking activity in plants as mechanism usually underlying their spasmolytic effect [30,36,37]. To see, whether the relaxant effect of *A. spinosus* is mediated through the same mechanism, the tissue was pretreated with high K^+^ (80 mM), which produced a sustained contraction. The contractile response induced by high concentration of K^+^ (80 mM) is due to release and influx of extracellular Ca^++^ through L-type voltage-sensitive channels [37]. The plant extract completely relaxed the K^+^ (80 mM)-induced contraction in isolated rabbit jejunum indicating the Ca^++^ channel blocking activity. The presence of CCB activity in *A. spinosus* was further strengthened when the plant extract shifted the Ca^++^ CRCs towards right, similar to diltiazem, a standard Ca^++^ channel blocker [38]. The presence of spasmolytic activity in the plant mediated through CCB mechanism may be responsible for its medicinal use in abdominal colic and perhaps also meant by nature to offset the excessive gut stimulant action that is usually observed with chemical drugs.

The plant material has traditionally been used for the management of respiratory diseases like asthma and bronchitis. To see the possible bronchodilator activity, the crude extract was tested on CCh-induced bronchospasm in anesthetized rats, where it caused a dose-dependent inhibition of CCh-induced bronchoconstriction, similar to aminophylline, a known bronchodilator agent [39]. To further explore the possible mechanism in its bronchodilator activity, the plant extract was tested on rabbit tracheal preparations precontracted with high K^+^ or CCh. The crude extract completely relaxed high K^+^ and CCh-induced contractions at similar concentration, whereas, diltiazem was more potent against K^+^ induced contraction, a typical characteristic of a pure CCB [40]. The CCB component in *A. spinosus* was confirmed when it shifted the Ca^++^ CRCs constructed in rabbit trachea towards right, similar to diltiazem. The Ca^++^ channel blockers have been found to be useful in bronchospastic disorders [40,41]. To see, if the bronchodilator effect of the crude extract was mediated through β_2_-agonist activity, inhibitory effect of the plant extract on CCh-induced spasms was tested in the presence of propranolol (1 μM), a non-specific β–adrenergic receptor antagonist [42]. In the presence of propranolol, the relaxant effect of *A. spinosus* extract on CCh-induced contraction was observed at significantly higher concentration, indicating the presence of β–adrenergic activity. The β_2_–adrenergic agonists are widely used in clinical practice as bronchodilator agents [42].

Interestingly, the crude extract of *A. spinosus* increased force of contraction followed by relaxation at the higher concentration. The cardio-tonic effect was diminished in tissues pretreated with propranolol and the relaxant effect became more prominent. The cardio-stimulant effect of adrenergic drugs is mediated through β_1_-adrenergic receptors, whereas is bronchodilator effect through β_2_ adrenergic receptors [42]. Presence of a combination of cardio-stimulant and bronchodilator activity, suppressed by propranolol, confirmed the presence of non-specific β-adrenergic agonist activity similar to that of isoprenaline. Isoprenaline, though very effective bronchodilator, but has limited clinical use due to associated cardiac stimulation as side-effect [42]. Interestingly, when tested on heart, there was mild ionotropic effect but no significant increase was observed on hear rate at low dose, while inhibitory effect was observed at higher doses. This is perhaps because of co-existence of cardiac stimulant (β_1_) and cardiac depressant activity due to CCB constituents, which has opposing effect in the heart, while additive and/or synergistic interactions with bronchodilator activity, a desired effect in air ways disorders. The β-adrenergic activity of *A. spinosus* was not seen in jejunal preparations that may be due to the cardiac and bronchial tissue selectivity of β-adrenergic effect.

Activity-directed fractionation revealed that the spasmolytic effect of the crude extract of *A. spinosus* was mainly concentrated in the ethyl acetate fraction which caused inhibition of spontaneous as well as K^+^-induced contraction, whereas the spasmogenic effect was concentrated in the aqueous fraction along with a weak spasmolytic activity. This pattern of separating biological activities among the fractions is in accordance with our previous findings, that the spasmogenic activity of crude plant extracts is usually concentrated in the aqueous fractions while the spasmolytic component is concentrated in organic fractions [43,44]. We also speculate that the spasmogenic component of the plant may not get absorbed systemically due to its polar nature as reflected by its water solubility and would not be able to interfere with its bronchodilator activity.

## Conclusion

These data, suggesting the presence of laxative effect in the plant mediated partly through cholinergic action, spasmolytic effect through CCB activity and bronchodilator activity through a combination of CCB and β-agonistic activities, explain the medicinal use of *A. spinosus* in constipation, abdominal colic and airways disorders. Such coexistence of β-agonist activity with CCB constituents offers synergistic and side-effect neutralizing potential, a typical characteristic of natural products [45].

## Abbreviations

ACh: Acetylcholine; *A. spinosus*: *Amaranthus spinosus*; As.Cr: The crude extract of *A. spinosus*; As.EtAc: The ethyl acetate fraction of the crude extract of *A. spinosus*; As.Aq: The aqueous fractions of the crude extract of *A. spinosus*; CCB: Ca^++^ channel blockade; CCh: Carbachol; CI: Confidence interval; CRCs: Concentration-response curves; EC_50_: Median effective concentration; n: Number of experiments; VDLCs: Voltage dependent L-type Ca^++^ channels.

## Competing interests

The authors declare that they have no competing interests.

## Authors’ contributions

AHG and SB designed the project and supervised the study. MAC carried out the experimental work, data analysis, literature search and drafted manuscript. MHM and NR helped in study design, analysis of data and preparing draft manuscript. II also helped in the preparation of draft manuscript. All authors read and approved the final manuscript for publication.

## Pre-publication history

The pre-publication history for this paper can be accessed here:

http://www.biomedcentral.com/1472-6882/12/166/prepub
